# Increased Mitochondrial Calcium Fluxes in Hypertrophic Right Ventricular Cardiomyocytes from a Rat Model of Pulmonary Artery Hypertension

**DOI:** 10.3390/life13020540

**Published:** 2023-02-15

**Authors:** Anna Maria Krstic, Amelia S. Power, Marie-Louise Ward

**Affiliations:** Department of Physiology Faculty of Medical and Health Sciences, University of Auckland, Auckland 1142, New Zealand

**Keywords:** cardiomyocytes, calcium transients, mitochondrial calcium fluxes, hypertrophy, monocrotaline, stimulated emission depletion (STED) microscopy

## Abstract

**Simple Summary:**

In pulmonary artery hypertension, right ventricular (RV) afterload is increased, which requires the cardiomyocytes to contract with greater force against the additional pulmonary artery pressure. In response, RV cardiomyocytes increase contractile protein content to maintain greater workload, consuming larger amounts of energy (supplied by the mitochondria) on a beat-to-beat basis. Failing hearts have been described as an “engine out of fuel”, but it is unclear how the mitochondria match ATP supply to demand in hypertrophic hearts prior to failure. Therefore, our aims were (i) to measure beat-to-beat mitochondrial Ca^2+^ fluxes, and (ii) to determine mitochondrial abundance and function in hypertrophied cardiomyocytes prior to the onset of heart failure. To identify the early adaptive changes in energy supply prior to failure, we utilised a rat model of pulmonary artery hypertension to investigate RV cardiomyocytes during compensated hypertrophy in comparison to their normotensive controls. Mitochondrial Ca^2+^ fluxes were increased in hypertrophied cardiomyocytes, but no difference was found in oxidative phosphorylation between the groups. This suggests that the larger mitochondrial Ca^2+^ transients are a compensatory mechanism to match ATP supply to the increased energy demands of hypertrophic cardiomyocytes.

**Abstract:**

Pulmonary artery hypertension causes right ventricular hypertrophy which rapidly progresses to heart failure with underlying cardiac mitochondrial dysfunction. Prior to failure, there are alterations in cytosolic Ca^2+^ handling that might impact mitochondrial function in the compensatory phase of RV hypertrophy. Our aims, therefore, were (i) to measure beat-to-beat mitochondrial Ca^2+^ fluxes, and (ii) to determine mitochondrial abundance and function in non-failing, hypertrophic cardiomyocytes. Male Wistar rats were injected with either saline (CON) or monocrotaline (MCT) to induce pulmonary artery hypertension and RV hypertrophy after four weeks. Cytosolic Ca^2+^ ([Ca^2+^]_cyto_) transients were obtained in isolated right ventricular (RV) cardiomyocytes, and mitochondrial Ca^2+^ ([Ca^2+^]_mito_) was recorded in separate RV cardiomyocytes. The distribution and abundance of key proteins was determined using confocal and stimulated emission depletion (STED) microscopy. The RV mitochondrial function was also assessed in RV homogenates using oxygraphy. The MCT cardiomyocytes had increased area, larger [Ca^2+^]_cyto_ transients, increased Ca^2+^ store content, and faster trans-sarcolemmal Ca^2+^ extrusion relative to CON. The MCT cardiomyocytes also had larger [Ca^2+^]_mito_ transients. STED images detected increased mitochondrial protein abundance (TOM20 clusters per μm^2^) in MCT, yet no difference was found when comparing mitochondrial respiration and membrane potential between the groups. We suggest that the larger [Ca^2+^]_mito_ transients compensate to match ATP supply to the increased energy demands of hypertrophic cardiomyocytes.

## 1. Introduction

In pulmonary artery hypertension (PAH), right ventricular (RV) afterload is increased, which requires RV cardiomyocytes to contract with greater force to eject a sufficient stroke volume. In response, RV cardiomyocytes hypertrophy, thus increasing their contractile protein content. This allows the RV to contract against greater pressures [1]. Due to this chronic increase in workload, hypertrophic hearts constantly require larger amounts of ATP to achieve an adequate cardiac output on a beat-to-beat basis. We have previously shown that, when RV hypertrophy progresses to failure, there is significant mitochondrial dysfunction [2,3]; however there are changes in Ca^2+^ handling that precede failure [4,5,6], which can affect both energy supply and force development. Previous evaluation of cardiomyocyte ATP, ADP, and P_i_ in vivo has shown that their concentrations remain relatively constant over a wide range of cardiac outputs [7]. The mitochondria play an important role in matching ATP supply to meet the ever-changing demands of the heart by utilising two key regulatory mechanisms. The first is an ADP metabolic feedback mechanism [8], and the second senses the magnitude of cytosolic Ca^2+^ changes during EC coupling [9]. Ca^2+^ can diffuse from the cytosol into the mitochondria via the voltage-dependent anion channel 1 on the outer mitochondrial membrane [10], where it then gets taken up into the mitochondrial matrix via the mitochondrial Ca^2+^ uniporter (MCU) on the inner mitochondrial membrane [11]. Ca^2+^ can then enhance the activity of various Ca^2+^ sensitive enzymes of the Krebs cycle, ultimately increasing ATP production [12]. Extrusion of mitochondrial Ca^2+^ occurs via the mitochondrial Na^+^/Li^+^/Ca^2+^ exchanger (mNLCX), which prevents mitochondrial Ca^2+^ overload and the pathological opening of the mitochondrial transition pores (mPTP) [13]. It is known that the mitochondria undergo a number of changes in the failing heart (for a review see Xu et al [14], one of which is attributed to Ca^2+^ overload [15]. It was thought that increased MCU Ca^2+^ re-uptake during relaxation could be protective by reducing arrhythmias caused by elevated levels of [Ca^2+^]_cyto_ [16]. On the other hand, high levels of [Ca^2+^]_mito_ can also stimulate the opening of the mPTP, which can cause significant damage to the mitochondria and disrupt ATP production, interfering with energy supply-and-demand matching [13]. Nonetheless, if the cardiomyocyte mitochondria cannot match the heart’s energetic demands, cardiac output can become compromised, which is a cardinal feature of heart failure (HF).

Prior to the onset of HF, there is an earlier state of compensatory cardiac hypertrophy. During compensated cardiac hypertrophy, cardiomyocytes require greater amounts of ATP to be produced per cell. However, it is currently unclear whether this stage is associated with an increased abundance of mitochondria per hypertrophied cell, or whether the mitochondria present are simply required to work harder to supply the ATP required. In addition, the kinetics of mitochondrial Ca^2+^ fluxes remain poorly understood, primarily due to the challenges of measuring mitochondrial Ca^2+^ in intact muscle preparations [17]. The main aim of our study was, therefore, to measure mitochondrial Ca^2+^ fluxes in healthy cardiomyocytes and also in hypertrophied cardiomyocytes prior to the onset of heart failure. Additionally, we aimed to determine whether cytosolic Ca^2+^ handling also had an impact on mitochondrial Ca^2+^ fluxes. Our second aim was to compare the distribution and relative abundance of the myofilaments, mitochondria, and ryanodine receptors between groups using confocal and stimulated emission depletion (STED) microscopy of fixed RV tissue sections. Our final aim was to examine mitochondrial oxidative phosphorylation during compensated hypertrophy. To achieve our aims, we utilised the monocrotaline rat model of pulmonary artery hypertension (PAH) during compensated right ventricular hypertrophy and their normotensive controls [6,18].

## 2. Materials and Methods

### 2.1. Animal Model and Ethical Approval

Pulmonary artery hypertension was induced in male Wistar rats of body weight 306.4 ± 6.6 g (mean ± SEM) by subcutaneous injection of 60 mg kg^−1^ monocrotaline (MCT, Sigma Aldrich, Castle Hill, Australia). Control (CON) rats were injected with the same volume of sterile saline. Post injection, rats were monitored and weighed regularly, as previously described [18]. Approval for this research was provided by the University of Auckland Animal Ethics Committee (AEC: 001807 and 001412) in accordance with the Code of Ethical Conduct of The University of Auckland and the New Zealand Animal Welfare Act 1999.

### 2.2. Cell Isolation

On the day of experimentation (30 ± 2 days post injection), the rats were euthanised, and their hearts were removed, weighed, and rapidly cannulated via the aorta. Dissociation of quiescent, rod-shaped cardiomyocytes was carried out using standard enzymatic digestion, as previously described [17]. The livers and lungs were also removed, blotted, and weighed for subsequent morphometric analysis.

### 2.3. Loading of Ca^2+^ Indicators

Isolated RV myocytes from each heart were divided into aliquots for the different measurements carried out. For cytosolic Ca^2+^, cells were loaded with 10 µM Fura-2/AM (Invitrogen, Thermo Fisher Scientific, Waltham, MA, USA) dissolved in 20 µL dimethyl sulphoxide anhydrous (DMSO, ThermoFisher) with 20% pluronic Invitrogen (Scientific, Life Technologies NZ, Auckland, New Zealand) for 20 min at room temperature. Cells were then washed with 1 mM Ca^2+^ Tyrode’s solution for at least 10 min prior to imaging. Mitochondrial Ca^2+^ measurements were taken in cells loaded with di-hydroRhod-2 (dhRhod-2), as previously described [17]. Briefly, a single 50 µg vial of Rhod-2 indicator (Invitrogen, Scientific, Life Technologies NZ) was dissolved in DMSO and 20% pluronic in DMSO was added. The smallest possible amount of Na^+^ borohydride (reducing agent) was dissolved in 20 µL methanol, and 10 µL was added to the Rhod-2 vial. After 5–10 min, 1 mL of cell suspension was added to 5 µM dhRhod-2 and left for 1 h at 37 °C. Cells were then washed for at least 30 min prior to imaging.

### 2.4. Experimental Solutions and Protocols

Cytosolic Ca^2+^ transients were recorded in RV cardiomyocytes loaded with Fura-2 (as described in Section 2.3). Cells were field-stimulated at 1 Hz (room temperature) and continuously superfused with Tyrode’s solution containing (in mM): 140 NaCl, 4 KCl, 10 Hepes, 1 MgCl_2_, 10 Glucose, and 1 CaCl_2_ (all Sigma-Aldrich Co. Merck, Darmstadt, Germany). Myocytes were imaged using a 20× fluorescent objective lens (0.75 NA) and illuminated with alternating 340 nm and 380 nm excitation wavelengths every 5 ms using an Optoscan monochromator and a spectrofluorometric PMT-based system (Cairn, Faversham, the UK). Emitted 510 ± 15 nm fluorescence was acquired at 400 Hz using Acquisition Engine Software (Cairn, Faversham, the UK) from whole cells as a measure of cytosolic [Ca^2+^] fluxes. Myocytes were subjected to 1 Hz stimulation until steady state was achieved. At this point, both the flow and the stimulation were switched off, and a 20 mM bolus of caffeine (Sigma-Aldrich) in Tyrode’s solution was applied to the bath to determine Ca^2+^ store content and sarcolemmal NCX activity. Superfusion with caffeine-free Tyrode’s solution and stimulation at 1 Hz were then re-commenced. The response to β-adrenergic stimulation with 1 mM Ca^2+^ Tyrode’s solution containing 1 µM isoproterenol (ISO, Sigma-Aldrich, cat no. 16504) was then determined.

Mitochondrial Ca^2+^ measurements were taken from dhRhod-2 loaded myocytes using the same spectrofluorometric system described above. Cells were field-stimulated at 0.1, 0.5, and 1 Hz (room temperature) and continuously superfused with 1.5 mM [Ca^2+^] Tyrode’s solution containing 1 µM isoproterenol and 150 µM spermine (Cayman Chemical, Ann Arbor, MI, USA, cat no. 136587-13-8). Myocytes were illuminated with a 542 ± 10 nm excitation wavelength and emitted fluorescence was collected at 581 nm (±10 nm) from whole cells as a measure of mitochondrial Ca^2+^ fluxes.

### 2.5. Labelling, Fixation, and Imaging of RV Tissue Sections

For details on fixation and labelling of isolated cardiomyocytes and tissue sections, refer to the Supplementary Data file. Confocal and STED images of RV sections dual-labelled with translocator of the outer mitochondrial membrane/ryanodine receptors (TOM20/RyR2) were obtained with an Olympus IX83 Abberior Facility Line STED microscope using a 60× oil immersion objective lens (NA 1.42). To show a larger portion of the tissue being analysed, confocal images were first captured with a 70 µm × 70 µm frame size at 80 nm pixel resolution. Then, both confocal and STED images were captured from a smaller portion of the tissue section (15 µm × 15 µm frame size) at a 15 nm pixel resolution with 594 nm and 640 nm lasers simultaneously at excitation laser powers 3–6% for confocal and STED images. Power for the STED depletion laser, emitted at 775 nm, was between 6% and 10%. Furthermore, confocal images of RV sections co-labelled for F-actin (Alexa Fluor 488 Phalloidin conjugate, 1:50, A12379, Thermofisher Scientific, Waltham, MA, USA) and mitochondria (TOM20) were obtained with a Zeiss LSM800 laser-scanning confocal microscope using a 63× oil-immersion objective lens (NA of 1.4). Images were captured at a 50 nm pixel resolution with 488 nm and 594 nm lasers simultaneously at 0.4% laser power.

### 2.6. Mitochondrial Respiration and Membrane Potential

Mitochondrial respiration and membrane potential (Δ*Ψ*) were measured in a separate cohort of saline and monocrotaline-injected rats (300–350 g; *n* = 20; AEC: R1403). Following rat euthanasia and cardiac excision, an RV sample was processed for high-resolution respirometry coupled to fluorometry with an Oxygraph-O2K™ respirometer (Oroboros™, Innsbruck, Austria). Approximately 20 mg of tissue was homogenised for 10 s in 25 volumes of ice-cold respiration buffer (RB; containing (in mM): MgCl_2_.6H_2_O (3), K-lactobionate (60), taurine (20), KH_2_PO_4_ (10), HEPES (20) and sucrose (110), with essential fatty acid free bovine serum albumin (BSA; 1 g L^−1^) and adjusted to pH 7.1 with KOH at 37 °C.). RV homogenate (1 mg mL^−1^) was added in duplicate to each chamber of the oxygraph containing 2 mL of RB prior to the addition of 2 µM safranine-O. Δ*Ψ* was measured by following safranine-O fluorescence using a filter set for excitation/emission wavelengths of 495/587 nm in the oxygraph fluorometers [19]. Complex I substrates were added and left to achieve a stable Δ*Ψ*. Oxidative phosphorylation (OXPHOS) was then initiated by the addition of 0.1 mM ADP along with 5 mM glucose and 2 U mL^−1^ of hexokinase to keep mitochondria in a phosphorylating state [20]. Then, 0.3 mM CaCl_2_ was added to each chamber followed by 0.25 mM titrations to achieve free [Ca^2+^] from 0.39 µM to 52 µM (calculated using the MAXCHELATOR CaMgADPEGTA program). Then, 2 µM carbonyl cyanide p- (trifluoro-methoxy) phenyl-hydrazone (FCCP) was added to achieve a zero-membrane potential for safranine-O calibration. To calculate the Δ*Ψ*, the Nernst equation (Equation (3)) was used as previously described [21]. Here, *R* is the gas constant, *F* is the Faraday constant, *T* is the temperature in Kelvin, and *z* is the valence state of safranine-O (+1). *C_in_* is the concentration of safranine-O within the mitochondria and *C_out_* is the concentration of safranine-O in the *RB*. *C_RB_* is the concentration of safranine-O measured in the RB at any point during the assay. Non-mitochondrial safranine-O uptake was subtracted from the total concentration of safranine-O (*C_total_* − *C_FCCP_*) determined by the signal following the addition of *FCCP*, which did not return to baseline which is normally seen with isolated mitochondria [21]. *V_RB_* is the volume of respiration buffer (2 mL), and *V_mito_* is the mitochondrial volume, which was estimated to be 3.1 μL mg^−1^ based on our previous assumptions [22].

(1) $$\Delta \Psi =\frac{RT}{zF}\ln\frac{C_{out}}{C_{in}}$$

(2) $$C_{out}=C_{RB}$$

(3) $$C_{in}=\frac{\left(C_{FCCP}-C_{RB}\right)V_{RB}}{V_{mito}}$$

### 2.7. Data Analysis

For further details of our data analysis, refer to the Supplementary Data file. Briefly, morphometric data were analysed by unpaired, two-tailed, *t*-tests to determine the differences in body and organ weights between groups. Cytosolic and mitochondrial Ca^2+^ transient data from RV myocytes were acquired using the Acquisition Engine (Cairn Research, Faversham, UK), and subsequently analysed using a custom-written IDL program (IDL version 6.2, Research Systems Inc., Boulder, CO, USA) to determine the various Ca^2+^ transient parameters. Parameters were subsequently analysed using two-way ANOVA for multiple comparisons between groups and interventions. Additionally, confocal images of RV sections co-labelled with phalloidin and TOM20 were acquired on Zen Blue software and subsequently analysed on Image J FIJI. Data from these images were also statistically analysed using a two-way ANOVA for multiple comparisons between groups (MCT vs. CON) and between labels (phalloidin vs. TOM20). Simultaneous confocal and STED images of RV sections immunolabelled for TOM20 and RyR2 were acquired using Lightbox Software (Abberior Instruments, Göttingen, Germany). STED images of RV tissue sections were also analysed using ImageJ FIJI to determine (i) the area of single clusters and (ii) the number of visible clusters per 1 µm^2^. These parameters were subsequently analysed by unpaired, two-tailed *t*-tests between groups. All statistical tests were performed using GraphPad Prism 9 Analysis software. The number of animals or hearts investigated is presented as “N”, while the number of cardiomyocytes is presented as “n”, unless otherwise stated.

## 3. Results

### 3.1. Evidence of Hypertrophy from Morphometric Data

**Figure 1:**
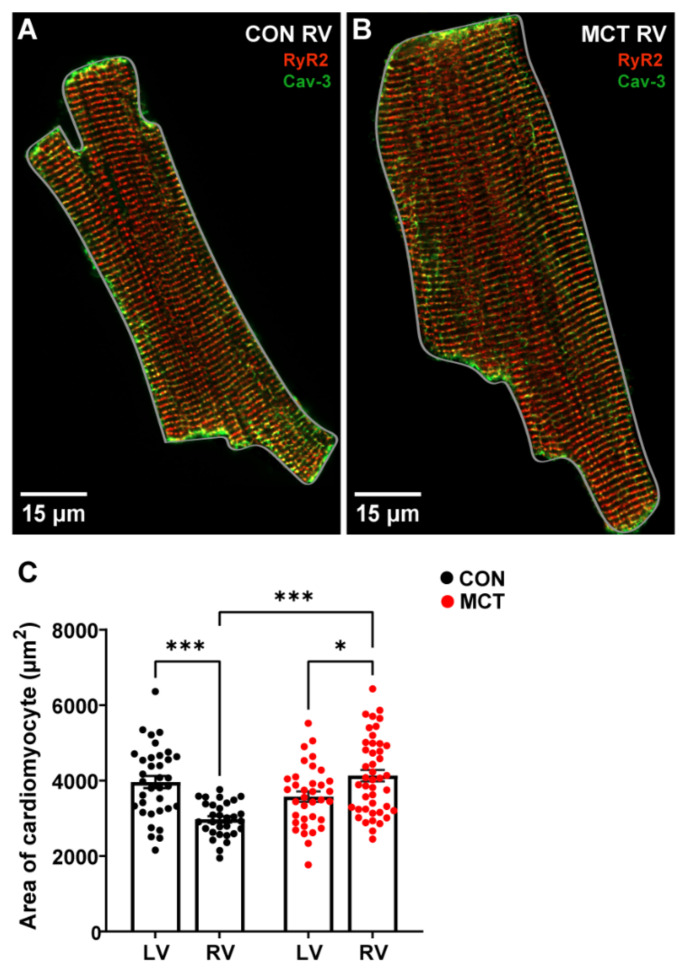
Area of left ventricular (LV) and right ventricular (RV) cardiomyocytes isolated from control (CON) and monocrotaline (MCT) rat hearts. (**A**,**B**) show fixed isolated RV cardiomyocytes from CON (**A**) and MCT (**B**) hearts immunolabelled for ryanodine receptors (RyR2, red) and Caveolin-3 (Cav-3, green). The grey outlines around the cardiomyocytes in (**A**,**B**) show how the cardiomyocyte area was measured. The myocyte in (**A**) (CON) had an area of 2047.20 μm^2^, whereas the hypertrophied myocyte in (**B**) (MCT) had an area of 3503.05 μm^2^. Data in (**C**) show cardiomyocyte areas from CON LV and RV cardiomyocytes (black) and MCT LV and RV cardiomyocytes (red). Bar graphs show mean ± SEM areas. N = 3 CON hearts with *n* = 35 LV, and *n* = 34 RV myocytes. N = 3 MCT hearts, with *n* = 35 LV, and *n* = 44 RV myocytes. * *p* < 0.05, *** *p* < 0.001.

At 30 ± 2 days (mean ± SEM) post injection, CON and MCT rats were sacrificed, and morphometric measurements were obtained. Table 1 shows that the CON body weights were higher than the MCT body weights on the day of experimentation (*p* < 0.001). The MCT animals had higher wet/dry lung weights compared to the CON animals (*p* < 0.01), with no difference in wet/dry liver weights, heart weight, or tibial length between groups. The heart weight:body weight (%) was not different between CON and MCT animals (*p* = 0.06). The cardiomyocyte area was calculated by measuring the perimeter of isolated cells, as illustrated by the grey outlines around representative cardiomyocytes in Figure 1A,B. Figure 1C shows that the LV CON myocytes were larger (mean ± SEM, 3963.54 ± 159.66 μm^2^) relative to the CON RV myocytes (3252.55 ± 152.08 μm^2^, *p* < 0.01). However, the MCT RV myocyte area (4134.29 ± 152.56 μm^2^) was higher than both the CON RV (*p* < 0.001) and the MCT LV myocyte areas (3578.31 ± 137.93 μm^2^, *p* < 0.05). There were no differences between CON and MCT LV myocytes or CON LV myocytes vs. MCT RV myocytes.

### 3.2. Response to β-Adrenergic Stimulation

**Figure 2:**
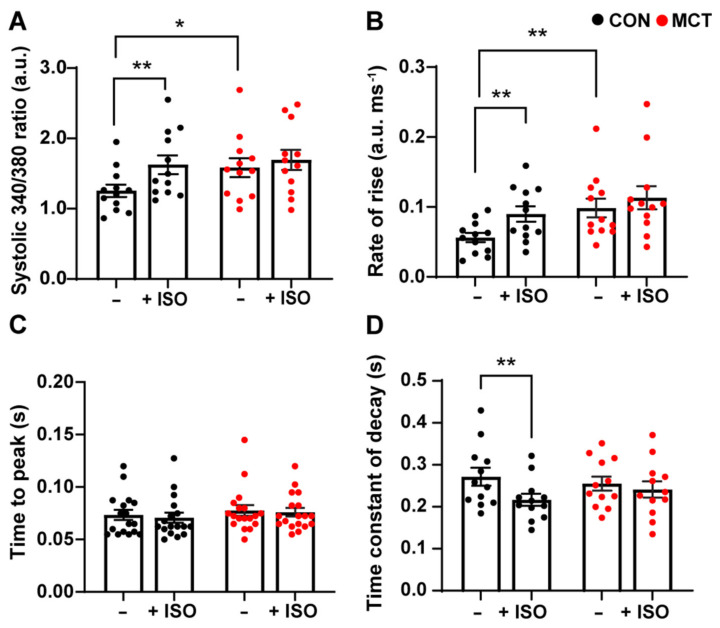
Cytosolic Ca^2+^ response of control (CON, black) and monocrotaline (MCT, red) right ventricular (RV) cardiomyocytes to 1 µM isoproterenol. Myocytes were stimulated at 1 Hz and super-fused with 1 mM Ca^2+^ Tyrode’s solution before (–) and during isoproterenol (+ISO). (**A**) shows peak systolic 340/380 ratio, while (**B**,**C**) show maximum rate-of-rise, and time-to-peak 340/380 fluorescence, respectively. The time constant of fluorescence decay is shown in (**D**). Results are presented as mean ± SEM, *n* = 12 RV myocytes from N = 4 CON hearts, and *n* = 12 RV myocytes from N = 4 MCT hearts. * *p* < 0.05, ** *p* < 0.01.

Cytosolic Ca^2+^ fluxes were measured in response to β-adrenergic stimulation in RV myocytes subjected to 1 µM isoproterenol (ISO). Figure 2 shows the mean ± SEM data recorded from the CON and MCT RV cardiomyocytes before ISO (−) and during response to ISO (+ISO). Figure 2A shows that ISO increased the amplitude of [Ca^2+^]_cyto_ transients for CON RV myocytes (mean ± SEM 340/380 ratio, 1.99 ± 0.15 a.u.) vs. before ISO (1.41 ± 0.14 a.u., *p* < 0.01), whereas MCT myocytes showed no change in [Ca^2+^]_cyto_ amplitude in response to ISO (1.67 ± 0.16 a.u.) relative to pre-ISO (1.51 ± 0.16 a.u.). However, MCT RV myocytes had larger baseline [Ca^2+^]_cyto_ transients (1.58 ± 0.46 a.u.) relative to CON RV myocytes (1.26 ± 0.31 a.u., *p* < 0.05) before the introduction of ISO. Figure 2B shows that CON RV myocyte [Ca^2+^]_cyto_ transients had faster maximum rate-of-rise in response to ISO (0.09 ± 0.01 a.u. ms^−1^) compared to pre-ISO (0.06 ± 0.01 a.u. ms^−1^, *p* < 0.01). MCT RV myocytes showed no change in maximum rate-of-rise in response to ISO; yet the maximum rate-of-rise before ISO was faster for MCT cardiomyocytes (0.10 ± 0.11 a.u. ms^−1^) in comparison to CON (0.06 ± 0.09 a.u. ms^−1^). In addition, neither CON nor MCT RV myocytes showed a change in the time-to-peak fluorescence in response to ISO (Figure 2C). ISO reduced the time constant of decay in CON RV myocytes (Figure 2D) from the baseline (0.27 ± 0.02 s) to (0.22 ± 0.014 s, *p* < 0.01), whereas ISO had no effect on the time constant of decay of MCT RV cardiomyocytes.

### 3.3. Sarcoplasmic Reticulum Ca^2+^ Store Content

**Figure 3:**
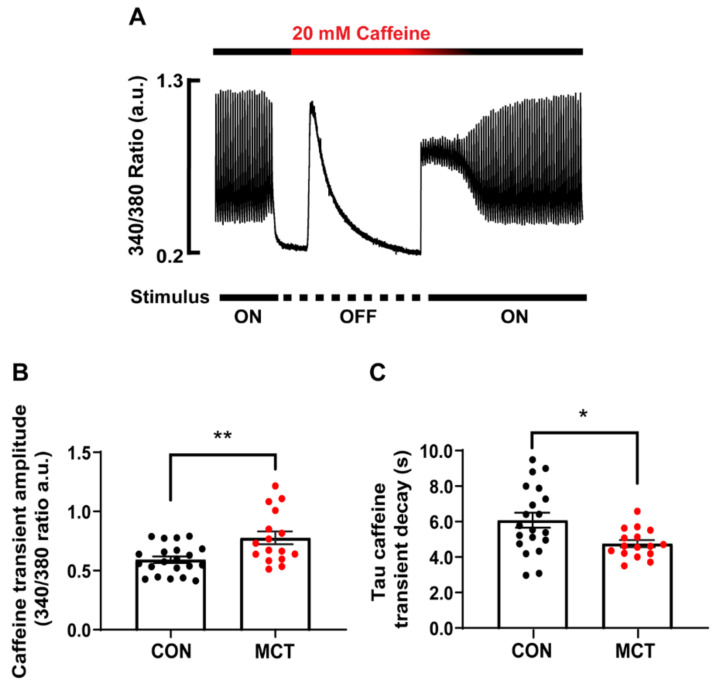
Ca^2+^ store content of isolated right ventricular (RV) cardiomyocytes from control (CON, black) and monocrotaline (MCT, red) rat hearts. (**A**) shows the response of a CON cardiomyocyte before, during and after application of 20 mM caffeine during the absence of stimulation. (**B**) shows mean ± SEM caffeine-induced Ca^2+^ transient amplitude of CON and MCT cardiomyocytes, while (**C**) shows the mean ± SEM time constant of caffeine-induced Ca^2+^ transient decay. Results are from *n* = 21 RV myocytes from N = 5 CON hearts, and *n* = 16 RV cardiomyocytes from N = 4 MCT hearts. * *p* < 0.05, ** *p* < 0.01.

SR Ca^2+^ store content was assessed by addition of a 20 mM caffeine bolus to the solution bathing the CON and MCT RV cardiomyocytes. Figure 3A shows a representative caffeine-induced [Ca^2+^]_cyto_ transient recorded from a single CON RV myocyte. The amplitude of the caffeine transient (340/380 ratio) in the absence of stimulation gave a measure of total [Ca^2+^]_SR_, while the time constant of the caffeine [Ca^2+^]_cyto_ transient decay in the continued presence of caffeine gave a measure of trans-sarcolemmal Ca^2+^ efflux. Figure 3B shows that the MCT RV myocytes had larger caffeine-induced [Ca^2+^]_cyto_ transients (0.78 ± 0.06 a.u.) in comparison to the CON RV myocytes (0.59 ± 0.03 a.u., *p* < 0.01). The MCT RV myocytes also showed a decreased time constant of decay (Figure 3C, 4.75 ± 0.20 s) relative to the CON RV myocytes (6.07 ± 0.42 s, *p* < 0.05).

### 3.4. Beat-to-Beat Mitochondrial Ca^2+^ Fluxes

**Figure 4:**
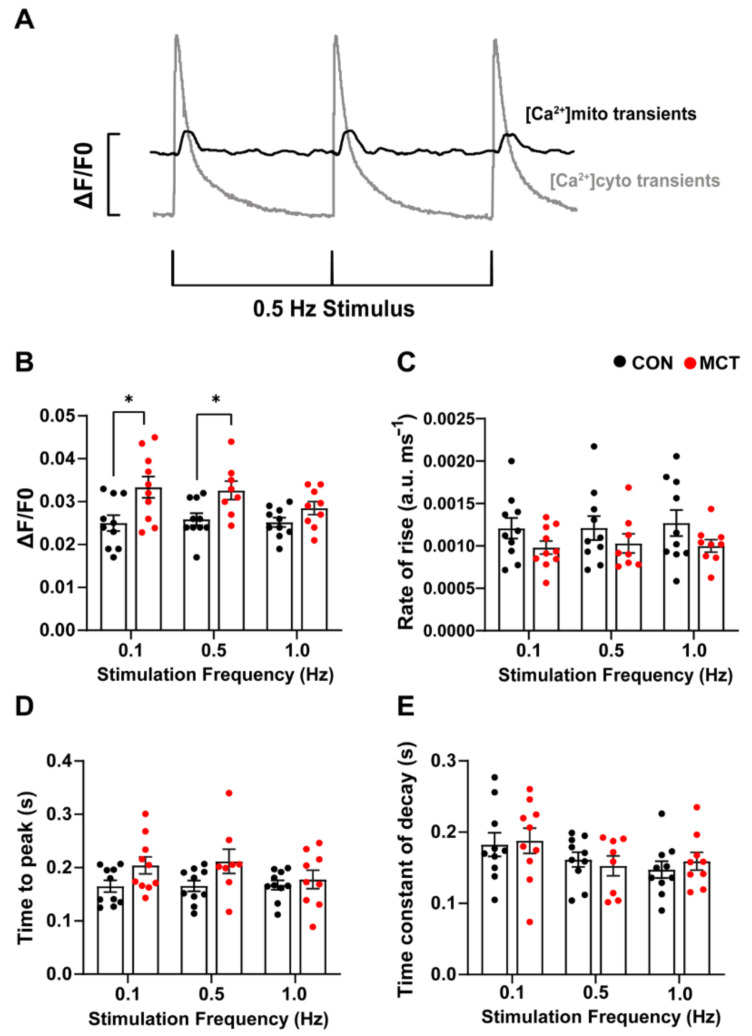
Response of mitochondrial Ca^2+^ transients to 0.1, 0.5, and 1 Hz stimulation frequencies obtained from control (CON, black) and monocrotaline (MCT, red) isolated right ventricular (RV) cardiomyocytes. Myocytes were continuously superfused with 1.5 mM Ca^2+^ Tyrode’s solution containing 1 µM isoproterenol and 150 µM spermine. (**A**) shows representative mitochondrial Ca^2+^ transients (black) superimposed with cytosolic Ca^2+^ transients (grey) from a separate cell in response to 0.5 Hz stimulation, to demonstrate differences in the time courses between cytosolic and mitochondrial Ca^2+^ measurements. (**B**,**C**) show the mean [Ca^2+^]_mito_ amplitude (ΔF/F0) and maximum rate-of-rise in fluorescence, respectively, between CON and MCT RV myocytes. (**D**) shows the time-to-peak for mitochondrial fluorescence, and (**E**) displays the mitochondrial time constant of decay. Results are expressed as mean ± SEM, *n* = 10 RV myocytes from N = 3 CON hearts, and *n* = 10 RV myocytes from N = 3 MCT hearts. * *p* < 0.05.

Beat-to-beat mitochondrial Ca^2+^ transients (Figure 4A) were recorded in the CON and MCT RV cardiomyocytes loaded with di-hydroRhod-2. The MCT RV cardiomyocytes had larger mitochondrial Ca^2+^ transient ([Ca^2+^]_mito_) amplitude (ΔF/F0, Figure 4B) at 0.1 Hz (mean ± SEM, 0.033 ± 0.002 a.u.) and 0.5 Hz stimulation (0.032 ± 0.002 a.u.) in comparison to the CON RV cardiomyocytes (0.1 Hz, 0.025 ± 0.002 a.u., *p* < 0.001 and 0.5 Hz 0.026 ± 0.001 a.u., *p* < 0.05). Figure 4C,D shows no change in the maximum rate-of-rise of [Ca^2+^]_mito_ and time-to-peak fluorescence in MCT RV myocytes relative to CON RV myocytes at any stimulation frequency. The MCT RV myocytes had a time-to-peak fluorescence of 0.201 ± 0.015 s at 0.1 Hz, and 0.206 ± 0.021 s at 0.5 Hz, relative to the CON RV myocytes, which had a time-to-peak fluorescence of 0.165 ± 0.011 s at 0.1 Hz and 0.166 ± 0.010 at 0.5 Hz (*p* = 0.15, Figure 4D). No difference in the time constant of fluorescence decay (Figure 4E) was found between groups, or between stimulation frequencies.

### 3.5. Mitochondrial Abundance in RV Fixed Tissue

**Figure 5:**
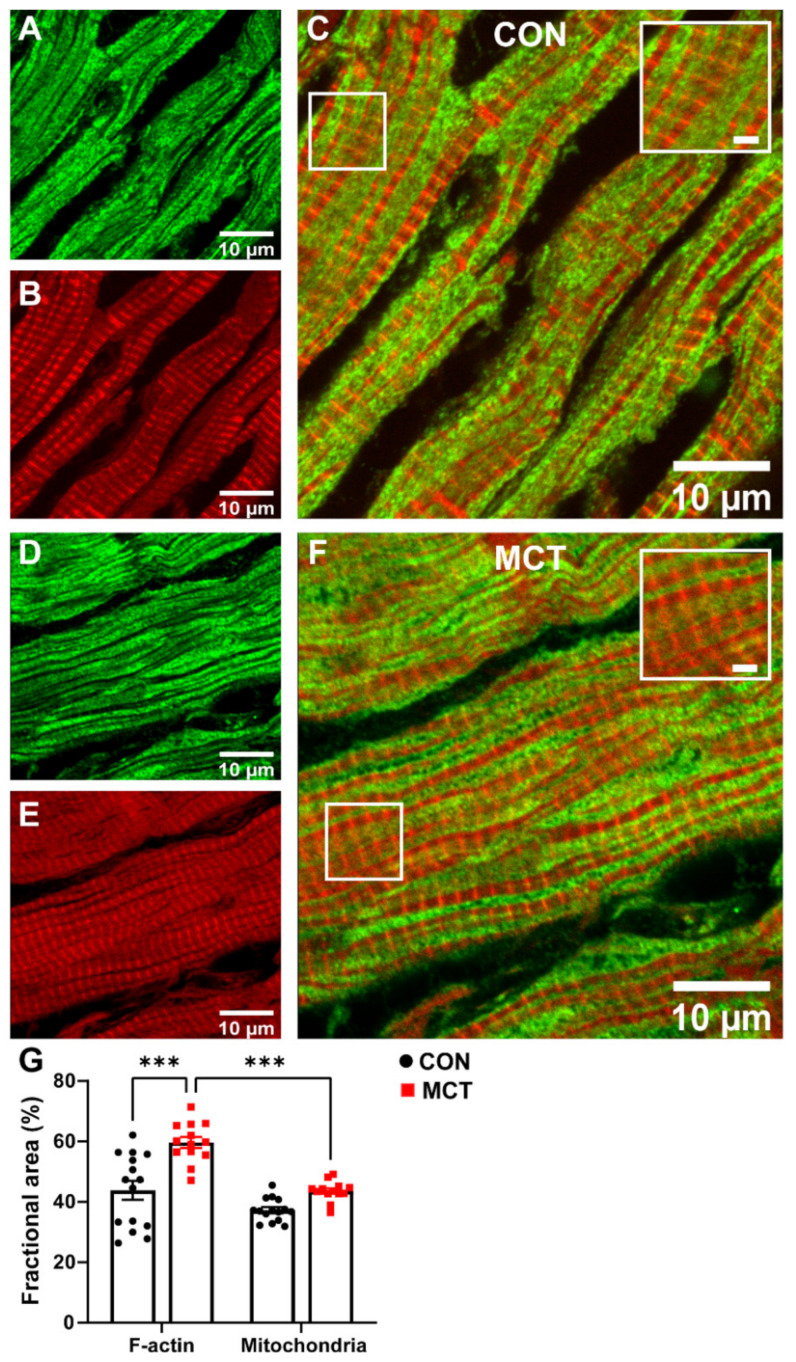
Representative confocal images of contractile protein and mitochondria in longitudinal right ventricular (RV) tissue sections. (**A**,**D**) show TOM20 labelling for mitochondria (green) in CON (**A**) and MCT (**D**), while (**B**,**E**) show phalloidin labelling for F-actin (red) in the same longitudinal sections in CON (**B**) and MCT (**E**). Images in (**C**,**F**) display superimposed TOM20 and phalloidin labelling in CON (**C**) and MCT (**F**) hearts. Scale bars of 2 µm are shown for the inset images within (**C**,**F**). The fractional area of the cardiomyocyte occupied by F-actin (i.e., % of phalloidin labelling) and mitochondria (i.e., % of TOM20 labelling) in CON (black) and MCT (red) RV tissue is presented in (**G**). Results are expressed as mean ± SEM from 3–5 50 µm × 50 µm sections/heart, for N = 3 CON and N = 3 MCT hearts. *** *p* < 0.001.

The area of cardiomyocyte occupied by mitochondria relative to the area occupied by the myofilaments was compared in longitudinal sections from healthy and hypertrophic RV tissue sections. Figure 5 shows confocal images of representative CON and MCT RV longitudinal sections co-labelled with phalloidin (red, for F-actin) and TOM20 (green, for mitochondria). Superimposed F-actin and TOM20 labelling of both CON (Figure 5C) and MCT (Figure 5F) RV tissue showed that TOM20 labelling was parallel to, and in close association with, F-actin labelling, indicating that the myofibrils were sandwiched between mitochondria. The mean data (Figure 5G) show the fractional area of TOM20 and phalloidin labelling in tissue sections from N = 3 CON and N = 3 MCT hearts. Figure 5G shows that the MCT RV sections had increased area of F-actin labelling per myocyte (59.7 ± 1.8%) relative to the CON sections (43.8 ± 3.1%, *p* < 0.001), with no difference in the mitochondrial area between CON (37.2 ± 1.0%) and MCT (43.6 ± 0.9%, *p* = 0.16) per myocyte. The ratio of the mitochondrial area relative to the F-actin area was therefore less for MCT RV myocytes (*p* < 0.001), whereas mitochondria and F-actin occupied equal areas of the myocytes in CON sections (*p* = 0.11).

**Figure 6:**
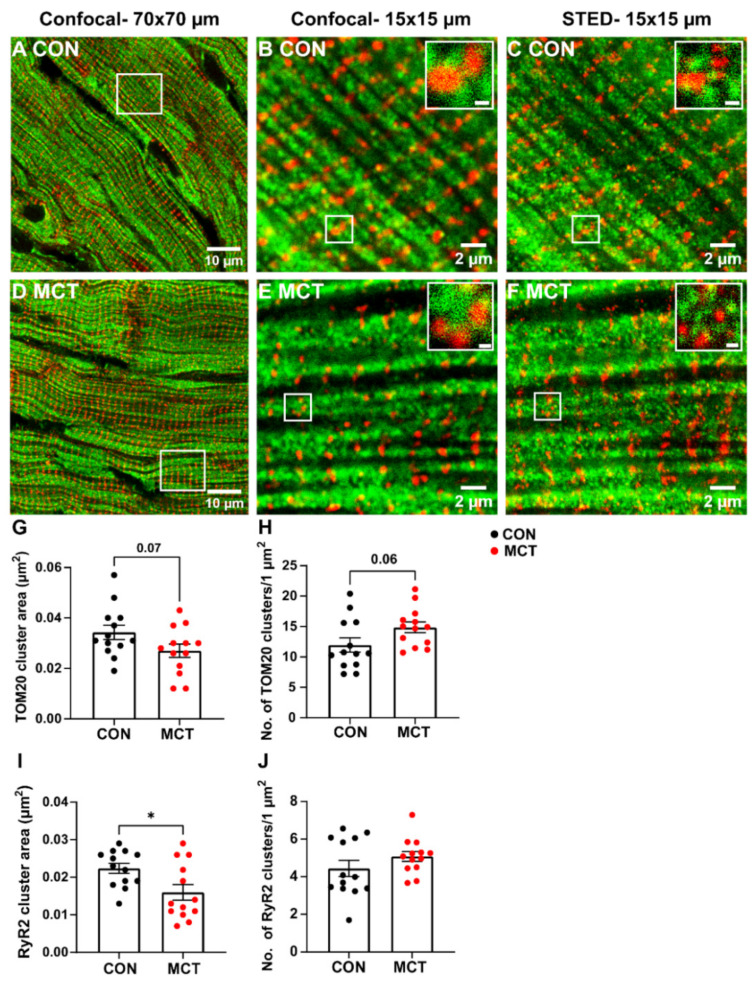
Confocal and stimulated emission depletion (STED) microscopy of RyR2 and TOM20 distribution in control (CON) and monocrotaline (MCT) right ventricular (RV) tissue sections. (**A**,**D**) are large-scale confocal images (70 µm × 70 µm frame size) showing control (CON, **A**) and monocrotaline (MCT, **D**) RV sections dual-labelled with TOM20 for mitochondria (green) and ryanodine receptors (RyR2, red). The white squares in (**A**,**D**) correspond to the small-scale confocal images (15 µm × 15 µm frame size) presented in (**B**,**E**) and the images displayed in (**C**,**F**), which were captured using STED microscopy. Scale bars of the insets in (**B**,**C**,**E**,**F**) are 200 nm. (**G**–**J**) show the mean data from STED images only. (**G**,**H**) indicate mean TOM20 cluster size, and the number of TOM20 clusters (per 1 µm^2^), respectively, in CON (black) and MCT (red) RV tissue. Likewise, (**I**,**J**) show the area and number of RyR2 clusters (per 1 µm^2^). Data are presented as mean ± SEM from N = 3 CON and N = 3 MCT hearts, and *n* = 3–5 RV sections/heart. * *p* < 0.05.

Mitochondrial and RyR2 distribution were also compared in longitudinal RV tissue sections between groups using confocal and STED microscopy (Figure 6). The large-scale confocal images show the longitudinal mitochondria (TOM20) and transverse distribution of the RyR2 in both CON and MCT tissue (Figure 6A,D), which was also evident in small-scale confocal and STED images (Figure 6B,C,E,F). However, the STED images also showed an increased number of TOM20 and RyR2 clusters (Figure 6C,F) in comparison to the confocal images for both groups (Figure 6B,E). Associations between RyR2 and TOM20 clusters were also evident in the small-scale STED images (Figure 6C,F). Mean data calculated from the STED images showed a trend towards decreased TOM20 cluster size in MCT RV myocytes (0.027 ± 0.01 µm^2^) relative to CON RV myocytes (0.034 ± 0.01 µm^2^, *p* = 0.07). Despite smaller TOM20 cluster sizes, the MCT RV myocytes had an increased number of TOM20 clusters per µm^2^ (Figure 6H, 14.9 ± 3.2 clusters/1 µm^2^ regions) vs. CON RV myocytes (11.2 ± 4.2 clusters/1 µm^2^ regions, *p* = 0.06). The MCT RV myocytes also showed smaller RyR2 cluster size (Figure 6I, 0.016 ± 0.007 µm^2^) in comparison to CON (0.022 ± 0.005 µm^2,^ *p* = 0.02) but with no difference in the number of RyR2 clusters per 1 µm^2^ between groups (Figure 6J, *p* = 0.2).

### 3.6. Mitochondrial Respiration and Membrane Potential

**Figure 7:**
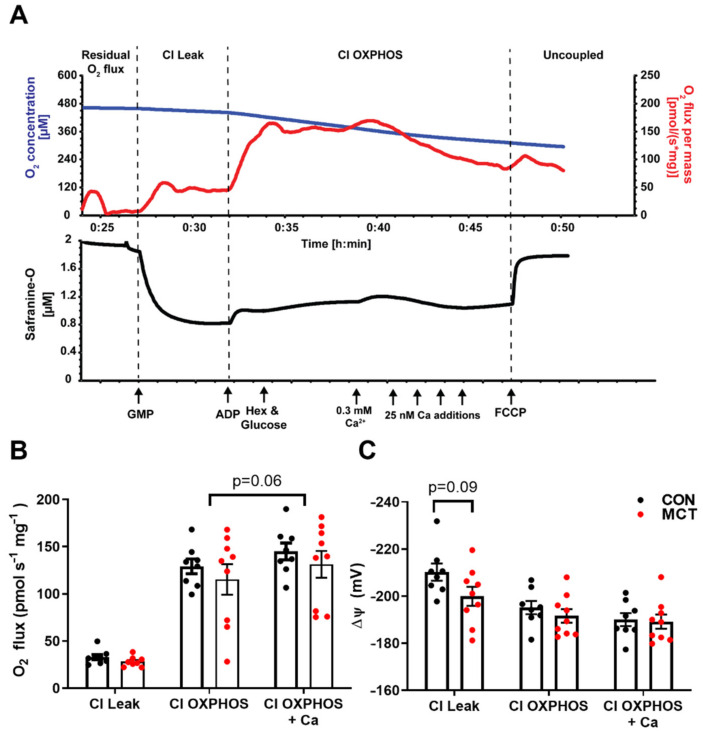
Mitochondrial respiration and membrane potential in control (CON) and monocrotaline (MCT) right ventricle homogenates. (**A**) is a representative oxygraph recording where the upper trace shows the change in O_2_ concentration (µM; blue) and the O_2_ flux (pmols s^–1^ mg^−1^; red) over time, and the lower trace shows concurrent measurement of safranine-O fluorescence (µM; black) which follows changes in mitochondrial membrane potential. Arrows indicate the addition of complex I substrates glutamate, pyruvate, and malate (GMP; CI Leak); 100 µM ADP with glucose and hexokinase (Hex) to keep the mitochondria in a phosphorylating state (CI OXPHOS); followed by additions of CaCl_2_ and, finally, the mitochondrial uncoupler carbonyl cyanide p- (trifluoro-methoxy) phenyl-hydrazone (FCCP). (**B**) shows steady-state O_2_ consumption (pmols s^–1^ m^–1^) for each respiratory state and at peak respiration with CaCl_2_, while (**C**) shows the corresponding mitochondrial membrane potential (mV) calculated from safranine-O fluorescence (see methods). Data are presented as mean ± SEM from N = 8 CON and N = 9 MCT hearts.

To test for any difference in mitochondrial function between CON and MCT hearts, concurrent measurements of O_2_ consumption and mitochondrial membrane potential were recorded in RV homogenates in the oxygraph. The driving force for ATP production via oxidative phosphorylation is from the electrochemical potential across the inner mitochondrial membrane, the majority of which is made up of the mitochondrial membrane potential (Δ*Ψ*). This was measured using Δ*Ψ* fluorescence indicator safranine-O with increasing free [Ca^2+^]. Figure 7A shows a representative trace from the control RV homogenate. Following the addition of CI substrates, a high Δ*Ψ* is developed which was partially depolarised following the addition of ADP, with hexokinase and glucose, which regenerates ADP to reach a steady-state Δ*Ψ*. An initial titration of 0.3 mM CaCl_2_ (free [Ca^2+^] of 0.39 μM) depolarises the Δ*Ψ* and stimulates the O_2_ flux; however, further titrations of CaCl_2_ result in the inhibition of O_2,_ and Δ*Ψ* repolarises until opening of the mitochondrial permeability transition pore (mPTP) and depolarisation of the Δ*Ψ*. This can be accelerated by the addition of FCCP. Mitochondrial respiration was not different between CON and MCT RV homogenates for all respiratory states before or after the addition of CaCl_2_ (Figure 7B). After the initial addition of CaCl_2,_ there was a small (non-significant *p* = 0.06) stimulatory effect of respiration. The developed Δ*Ψ* did not differ between the groups and the addition of ADP and CaCl_2_ similarly depolarised the Δ*Ψ*.

## 4. Discussion

### 4.1. Evidence of Hypertrophy from Morphometric Data

Changes in myocardial Ca^2+^ were studied in hypertrophic cardiomyocytes using the monocrotaline (MCT) rat model of pulmonary artery hypertension (PAH) and their saline-injected controls (CON). Morphometric data confirmed that MCT rats four weeks post-injection were not in heart failure, and there was no difference in heart weight to body weight between CON and MCT animals on the day of experimentation. This was unexpected as the hypertrophic RV was expected to increase the mass of the whole heart. However, LV atrophy has been previously reported in MCT myocardium [18,23], which can explain the lack of change in the HW:BW ratio between groups. Furthermore, measurements of cardiomyocyte area confirmed that isolated RV myocytes from MCT were hypertrophied with larger perimeters relative to CON RV and LV myocytes, and to MCT LV myocytes (Figure 1A–C).

### 4.2. Response to β-Adrenergic Stimulation

The response of RV cardiomyocytes to the non-selective β-AR agonist isoproterenol (ISO) showed that ISO increased peak systolic [Ca^2+^]_cyto_ in CON RV myocytes (Figure 2A) but not in MCT RV myocytes, although MCT RV myocytes had larger [Ca^2+^]_cyto_ transients in the absence of ISO relative to CON (Figure 2A). The time-to-peak fluorescence (Figure 2C) and diastolic [Ca^2+^]_cyto_ (Supplementary Figure S1) were unaffected by ISO in both groups, but only CON myocytes showed increased cytosolic Ca^2+^ transient kinetics during ISO (maximum rate-of-rise (Figure 2B) and time constant of decay (Figure 2D)). Overall, MCT myocytes did not show the expected response to β-AR stimulation [24,25]. The increased [Ca^2+^]_cyto_ transient amplitudes from MCT in comparison to CON (in the absence of ISO) is unlikely to be a result of endogenous β-AR activation in isolated cells stored in the physiological buffer following isolation. Instead, we suggest that the diminished MCT response to ISO might be a result of the SR Ca^2+^ stores already being at capacity prior to β-AR stimulation. A decreased response to ISO can also be due to the distribution of β-ARs, whereby spatially diverse cAMP diffusion occurs depending on the receptor subtype that is activated. For example, activation of β_1_-AR induces cell-wide cAMP diffusion and PKA activation, whereas the β_2_-AR subtype has been shown to be present specifically in the T-tubules, inducing a more localised cAMP diffusion/PKA activation [26]. MCT-induced RV hypertrophy is associated with T-tubular disorganisation [6,27], which might affect the distribution of β_2_-ARs [28], thereby altering cAMP diffusion and PKA activation of Ca^2+^ handling proteins localised to the T-tubules (i.e., the LTCC). The blunted response to ISO might also be due to either β-AR receptor downregulation or desensitisation, which has previously been reported in hypertrophic and failing MCT hearts [6,29,30].

### 4.3. Increased Ca^2+^ Store Content in Hypertrophy

Caffeine increases the opening probability of the RyR2, which makes it a useful tool for measuring the SR Ca^2+^ content of isolated cardiomyocytes. The continued presence of caffeine in the buffer solution prevents the accumulation of SR Ca^2+^ [31,32], while the sarcolemmal Ca^2+^ removal mechanisms eventually restore cytosolic [Ca^2+^] concentration to the diastolic level [33]. Therefore, subjecting myocytes to prolonged caffeine provided measurements of both total SR Ca^2+^ store content (amplitude) and NCX trans-sarcolemmal Ca^2+^ extrusion (from the time constant of the caffeine transient decay, Figure 3A). NCX accounts for the bulk of trans-sarcolemmal Ca^2+^ extrusion, and, in the rat, only ~5–10% is normally extruded across the NCX, while MCU and Ca^2+^ ATPase also provide minor contributions [32]. Hypertrophic MCT RV myocytes had increased SR Ca^2+^ store content in comparison to the CON RV myocytes (Figure 3B), resulting in larger baseline Ca^2+^ transient amplitudes and faster rates-of-rise in comparison to the CON myocytes (Figure 2A,B and Figure 3B). Since measurements of cytosolic [Ca^2+^] were made using a ratiometric fluorescent indicator, the higher amplitude transients must, therefore, indicate an increased cytosolic Ca^2+^ concentration rather than merely due to the hypertrophied MCT cardiomyocytes being larger in size than CON. Caffeine-induced Ca^2+^ transients from MCT RV cardiomyocytes also had a decreased time constant of decay relative to CON RV myocytes (Figure 3C), providing evidence that trans-sarcolemmal Ca^2+^ extrusion rate (flux) via NCX activity was increased in the MCT.

### 4.4. Beat-to-Beat Mitochondrial Ca^2+^ Fluxes

Ca^2+^ uptake via the mitochondrial calcium uniporter (MCU) is one of the key regulators of ATP production in the myocardium [34]. The MCU is a low-affinity Ca^2+^ transporter on the inner mitochondrial membrane, which is activated at high [Ca^2+^]_cyto_, such as at the peak of the [Ca^2+^]_cyto_ transient during EC coupling [9]. An influx of Ca^2+^ into the mitochondrial matrix enhances the activity of various Ca^2+^ sensitive enzymes of the Krebs cycle, which ultimately promotes ATP production [12]. Mitochondrial Ca^2+^ concentration fluctuates on a beat-to-beat basis, i.e., mitochondrial “transients” [17], thus relaying the cellular energy requirements directly to match supply to demand. Mitochondrial transients have a slower time-to-peak and time constant of decay relative to cytosolic Ca^2+^ transients (Figure 4A). In addition, MCT RV cardiomyocytes had larger mitochondrial Ca^2+^ transient ([Ca^2+^]_mito_) amplitudes at 0.1 Hz and 0.5 Hz stimulation in comparison to CON RV cardiomyocytes (Figure 4B). These results indicate that hypertrophic cardiomyocytes have increased mitochondrial Ca^2+^ uptake, probably as a result of a higher [Ca^2+^]_cyto_/[Ca^2+^]_SR_ [35]. This is consistent with our cytosolic Ca^2+^ measurements from MCT RV myocytes (Figure 2A,B and Figure 3B). It has been suggested that hypertrophic cardiomyocytes have a compensatory mechanism whereby the mitochondria act as a buffer of cytosolic Ca^2+^, reducing the incidence of delay after depolarisations and spontaneous Ca^2+^ release events [36,37,38]. However, others have proposed that the contribution of the MCU to cytosolic Ca^2+^ removal is approximately 1% [32,39]. The decay of [Ca^2+^]_mito_ transients reported in the present study, and by others [40,41], suggests that the mitochondria only temporarily increase their Ca^2+^ content between myocyte contractions. Our findings showed no difference between groups in mitochondrial Ca^2+^ transient kinetics at the stimulation frequencies investigated (Figure 4C,D), including no changes to the time constant of decay (Figure 4E). These results suggest that mNLCX activity, which is the primary transporter for mitochondrial Ca^2+^ extrusion during the decay [42], was unchanged between CON and MCT RV myocytes.

### 4.5. Mitochondrial Abundance in RV Fixed Tissue

Cardiac hypertrophy is characterised by increased cell size due to a higher abundance of contractile proteins [1]. This means that hypertrophic cardiomyocytes are capable of increased work, which requires more energy than the healthy control cardiomyocytes. In the present study, confocal images showed that the fractional area of mitochondria calculated per RV tissue section was not different in MCT relative to CON, despite a 16% increase in myofilament content (Figure 5A–G), as previously described [2]. Figure 5C,F show that the longitudinal labelling pattern of F-actin (using phalloidin) was distributed in parallel to, and in close association with, the mitochondria (labelled with a marker for the translocator of the outer mitochondrial membrane; TOM20). TOM20 is a subunit of the large TOM protein complex located on the outer mitochondrial membrane, with linkages to the inner membrane. Its function is to translocate nuclear-encoded proteins from the cytosol destined for the mitochondria [43]. TOM20 is highly abundant on the outer membrane, making it difficult to resolve its exact distribution when imaging with a modality beyond the diffraction limit—i.e., with confocal microscopy [44]. While confocal images did not detect a change in mitochondrial abundance between CON and MCT RV tissue (Figure 5), we further investigated mitochondrial clusters using STED imaging, which enabled improved lateral resolution from ~250 nm to ~40–60 nm.

STED microscopy of fixed cardiac tissue enabled the size and number of individual TOM20 clusters to be identified (Figure 6). A clustered distribution of TOM20 has been previously reported in super-resolution imaging of different cell types, excluding cardiomyocytes [45,46,47,48]. In addition, it has been suggested that the cluster density of TOM20 is tightly regulated and correlated with both the activity and location of the mitochondria, and that the TOM20 distribution is finely tuned to match the energetic demands of the cell [44,47]. This would mean that, since hypertrophic cardiomyocytes are larger (Figure 1B,C) and have increased [Ca^2+^]_mito_ transients (Figure 4B), larger TOM20 cluster densities would also be expected. In the present study, STED microscopy showed a trend toward decreased TOM20 cluster size in MCT RV tissue when compared to the control (Figure 6G), suggesting fewer TOM20 receptors per cluster. On the other hand, MCT RV tissue also showed a trend towards an increased number of clusters per µm^2^ relative to CON RV tissue (Figure 6H). This contradicts the data presented in Figure 5G, which showed no absolute change in mitochondrial content between CON and MCT hearts, despite an increase in contractile protein content. This is most probably due to the lack of resolution with standard confocal microscopy. An increased number of TOM20 clusters could either mean that more mitochondria are present or that TOM20 expression is increased in hypertrophic tissue. However, TOM20 expression has previously been shown to be unaffected in pathological cardiac hypertrophy [49]. Therefore, an increase in the number of mitochondria present in hypertrophic RV tissue could explain this result. Additionally, while our STED data showed a 24% increase in TOM20 clusters in hypertrophic RV tissue (Figure 6H), our functional data also showed that mitochondrial Ca^2+^ uptake was increased by 33% in MCT RV myocytes relative to CON RV myocytes (Figure 4B). Although the STED data from the present study showed a trend towards increased TOM20 cluster numbers, we cannot determine whether the mitochondrial distribution of TOM20 differed between groups.

Furthermore, it was evident that there was some overlap between the mitochondria and the RyR2 Ca^2+^ release sites in Figure 6, which was expected as this enables the mitochondria to immediately sense changes [Ca^2+^]_cyto_ during EC coupling [50]. The present study showed reduced RyR2 cluster size in hypertrophic RV tissue relative to the controls (Figure 6I) which could be a sign of RyR2 cluster fragmentation as reported by Sheard et al. [51]. These data contradict findings from other studies investigating hypertrophic tissue using super resolution imaging techniques, which showed either no change in RyR2 signal density [52] or an increased mean RyR2 cluster density [53]. Despite the smaller RyR2 cluster areas determined in MCT RV tissue, there were also no differences in the number of RyR2 clusters per µm^2^ between groups (Figure 6J). This indicates a possible scattered distribution of the RyR2 clusters throughout the myocyte, increasing its “nearest neighbour distance” (see Supplementary Figure S2). However, functional data from CON and MCT isolated cells presented in this study show no change in the time-to-peak fluorescence (i.e., the time of L-Type Ca^2+^ channel activation following cardiomyocyte stimulation to RyR2 Ca^2+^ release, Figure 2C) between groups. This suggests that either sarcolemmal Ca^2+^ fluxes are upregulated in MCT RV myocytes or the SR volume of hypertrophic myocytes is more extensive, which could overcome any irregularities of the T-tubular network.

### 4.6. Mitochondrial Respiration and Membrane Potential

Since mitochondria are responsible for producing 95% of the ATP consumed by cardiomyocytes [54], they have been implicated in the energy deficits observed in models of pathological hypertrophy. Measurement of O_2_ flux in vitro by high-resolution respirometry is the gold standard measure for mitochondrial function. There was no difference in O_2_ flux measured in RV homogenates from CON or MCT hearts in any respiratory state measured. Therefore, although the MCT hearts had significant RV hypertrophy, their mitochondrial oxidative capacity was similar to the control per mg of tissue. Addition of Ca^2+^ to the RV homogenate had a slight stimulation of O_2_. Ca^2+^ uptake into the mitochondria through the MCU utilises the Δ*Ψ* and may stimulate respiration flux through the ETS [55], although, at this concentration of free Ca^2+^ (0.39 µM) it is likely that any uptake would be through other uptake pathways that are not fully resolved [56]. The stimulatory effect of Ca^2+^ was similar for both MCT and control RV homogenates, suggesting that the mechanisms of Ca^2+^ uptake are unchanged between the groups. The Ca^2+^ transient is responsible for eliciting contraction and, in parallel, stimulates mitochondria [55]. However, prolonged mitochondrial exposure to high [Ca^2+^] triggers opening of the mPTP and irreversible depolarisation of the Δ*Ψ*, causing cardiomyocyte death [20]. Unfortunately, for these in vitro mitochondrial assays, we could not mimic the oscillating Ca^2+^ conditions observed in vivo, and titration of Ca^2+^ above ~0.5 µM resulted in gradual inhibition of respiration before triggering the mPTP. Therefore, only one bolus of 0.3 mM CaCl_2_ was used in these experiments which results in a free Ca^2+^ concentration of 0.39 µM. We calculated Δ*Ψ* from the uptake of fluorescent cation safranine-O which has been used previously [19,21,22]. Although it is documented to have some inhibitory effects on respiration [19], these are minimal at low safranine-O concentrations (2 µM) and are far less compared to routinely used Δ*Ψ* indicators such as JC-1 [57] and TPP+ [58]. It could also be assumed that any inhibitory effects of safranine-O are the same for the control and MCT. The maximum Δ*Ψ* that can be achieved in energized mitochondria is in the Leak state when no ADP is present. There was a slight (NS) depolarisation of the Δ*Ψ* measured in MCT RV homogenate in the Leak state compared to the controls (CON: −210 ± 4 mV, MCT: −200 ± 4 mV, *p* = 0.09), suggesting that MCT RV mitochondria have a lower driving force for ATP production. However, in the phosphorylating OXPHOS state, there was no difference between groups. Given that MCT hearts will have greater energy demand due to their increased myofilament area, these data support the idea that increased mitochondrial energy supply is met by enhanced beat-to-beat mitochondrial Ca^2+^ uptake rather than by increased mitochondrial capacity.

## 5. Conclusions

Overall, MCT RV cardiomyocytes showed increased cytosolic Ca^2+^ fluxes with a blunted response to β-adrenergic stimulation, and an increased SR Ca^2+^ store content. In addition, MCT RV cardiomyocytes also had larger beat-to-beat mitochondrial Ca^2+^ fluxes, which, we speculate, indicates a compensatory mechanism developed to match ATP supply to the greater energetic demands of the hypertrophic myocytes. While confocal images confirmed an increase in contractile protein content in MCT RV tissue sections, STED microscopy also revealed an increased number of TOM20 clusters, which, in some regions, were closely associated with the RyR2 clusters. Our data show—for the first time—evidence of a link between augmented [Ca^2+^]_mito_ uptake and increased mitochondrial abundance in hypertrophic cardiomyocytes. We conclude that this provides a potential mechanism by which hypertrophied cells compensate to match ATP supply to the increased energetic demands of additional contractile protein and bigger Ca^2+^ transients, prior to progression to heart failure.

## Figures and Tables

**Table 1:** Morphometric data from CON and MCT rats obtained on the day of experimentation. Heart weight:body weight (HW:BW %). Results are presented as mean ± SEM. N = number of rats/group. ** *p* < 0.01, *** *p* < 0.001.

	CON (N = 10)	MCT (N = 10)
Body Weight (g)	424.10 ± 7.85	375.10 ± 6.54 ***
Lung Weight (dry, g)	0.34 ± 0.01	0.45 ± 0.03 **
Lung Weight (wet, g)	1.49 ± 0.06	2.03 ± 0.10 ***
Liver Weight (dry, g)	6.96 ± 0.58	5.72 ± 0.47
Liver Weight (wet, g)	14.50 ± 0.49	13.95 ± 0.52
Heart Weight (g)	1.82 ± 0.07	1.85 ± 0.09
Tibial Length (mm)	53.02 ± 1.32	50.99 ± 1.13
HW:BW (%)	0.43 ± 0.02	0.49 ± 0.03

## Data Availability

Data supporting reported results can be obtained on request.

## Supplementary Materials

The following supporting information can be downloaded at: https://www.mdpi.com/article/10.3390/life13020540/s1, Methods: Labelling and fixation of tissue; Data Analysis; Figure S1: Diastolic response to isoproterenol; Figure S2: Distances between clusters.

## Abbreviations

dhRhod-2	di-hydroRhod-2
ISO	Isoproterenol
MCT	Monocrotaline
MCU	Mitochondrial calcium uniporter
mNLCX	Mitochondrial sodium lithium calcium exchanger
PAH	Pulmonary artery hypertension
STED	Stimulated emission depletion
TOM20	Translocator of the outer mitochondrial membrane
