# Supplementary material for: Increased Mitochondrial Calcium Fluxes in Hypertrophic Right Ventricular Cardiomyocytes from a Rat Model of Pulmonary Artery Hypertension
Source: Life (Basel). 2023 Feb 15;13(2):540. doi: 10.3390/life13020540 (PMC9967871; doi:10.3390/life13020540)
Supplement: Supplementary file 1 [file life-13-00540-s001.zip › life-2142939-supplementary.pdf]

Methods- Supplementary Files

Labelling and fixation of tissue

CON and MCT RV tissue blocks were fixed in 2 % PFA (ProSciTech, cat no. C006) for 1 hour at 4˚C, and were subsequently washed in 10, 20 and 30 % sucrose solutions (Sigma Aldrich, NZ) in one-hour increments. Tissue blocks were placed in labelled cryomolds containing optimal cutting temperature compound (both from Tissue Tek®, Sakura Finetek Inc., Torrance, CA, USA), then snap-frozen in liquid nitrogen. Frozen tissue was cut into 10 µm sections using a cryostat and subsequently mounted onto microscope slides prior to antibody labelling. For STED imaging, tissue sections were incubated with the primary antibodies mouse anti-RyR2 (1:100) and rabbit anti-TOM20 (1:100, SC-11415, Santa Cruz Biotechnology) and incubated overnight at 4°C. Sections were then incubated with secondary antibodies goat anti-mouse Abberior Star Red (1:100, STRED-1001, Abberior, Germany) and goat anti-rabbit Alexa 594 (1:100, A32740, Thermofisher Scientific) for 2 hours at room temperature. After antibody incubation, tissue sections were mounted in 2,2'-thiodiethanol based embedding media (TDE-0, cat no. MMTDE-2005, Abberior, Germany).

Data analysis

The area of isolated RV and LV cardiomyocytes from both groups were analysed using a tracing tool on ImageJ to measure the perimeter of the cells at resting length (i.e., unstimulated). Cytosolic and mitochondrial Ca^{2+} transient data from RV CON and MCT myocytes was acquired using Acquisition Engine software (Cairn Research, U.K). For cytosolic Ca^{2+} transients, the following transient parameters were analysed: peak systolic and diastolic fluorescence (340/380 ratio, a.u.), the maximum rate-of-rise in fluorescence (a.u. ms^{-1}), time to peak fluorescence following stimulation (s), and the time constant of fluorescence decay (s). All parameters listed were also analysed for mitochondrial Ca^{2+} transients, with the exception of systolic and diastolic fluorescence, which was replaced with transient amplitude normalized to diastolic fluorescence ($\Delta F/F0$). The parameters listed for both cytosolic and mitochondrial Ca^{2+} fluxes were subsequently analysed using GraphPad Prism 9 Analysis software and compared using two-way ANOVA for multiple comparisons between groups and interventions.

Confocal images of RV sections co-labelled with phalloidin and TOM20 were acquired on Zen Blue software and subsequently analysed on Image J FIJI. Fractional area of the total cell area occupied by phalloidin and TOM20 labelling was measured by creating a mask for each channel, then calculating the % of the total cell area occupied by each label. This was done to determine contractile protein (phalloidin) and mitochondrial (TOM20) abundance in CON and MCT RV tissue. Data from these images were statistically analysed in GraphPad Prism 9 using a two-way ANOVA for multiple comparisons between groups (CON *vs.* MCT) and between labels (phalloidin *vs.* TOM20).

Confocal and STED images of RV sections were acquired simultaneously using Lightbox Software (Abberior Instruments, Germany) and subsequently analysed using ImageJ FIJI. STED images of RV tissue sections (15 x 15 μm) labelled for TOM20 and RyR2 were analysed for the following parameters: i) the number of single TOM20 or RyR2 clusters per 1 μm^2, ii) the area of single TOM20 or RyR2 clusters (in μm^2) and iii) the nearest neighbour distance (NND), which determines distance between single clusters (in μm). Using ImageJ, images of TOM20 and RyR2 were analysed separately, and smoothed by applying a Gaussian Blur filter (1.0). Then, a threshold was applied to create a mask, which revealed isolated clusters for subsequent cluster analysis. For TOM20 images, Phansalkar auto local threshold was applied, and for RyR2, the ISO data threshold was used. This allowed measurements of both single cluster area and number of clusters per unit area[2]. Furthermore, NND was also analysed using ImageJ, and the same filters/masks listed above (with respect to each label) were applied to all images. The NND was determined using the NND plugin on ImageJ, and for each image/label, the minimum NND was compared between groups. All STED data were statistically analysed in GraphPad Prism 9 using unpaired two-tailed t-tests for comparisons of RyR2 and TOM20 clusters between groups (CON *vs.* MCT). Statistical significance of all analyses mentioned above was determined by a P value of less than 0.05. *P < 0.05, **P < 0.01, *** P < 0.001.

Discussion- Supplementary Files

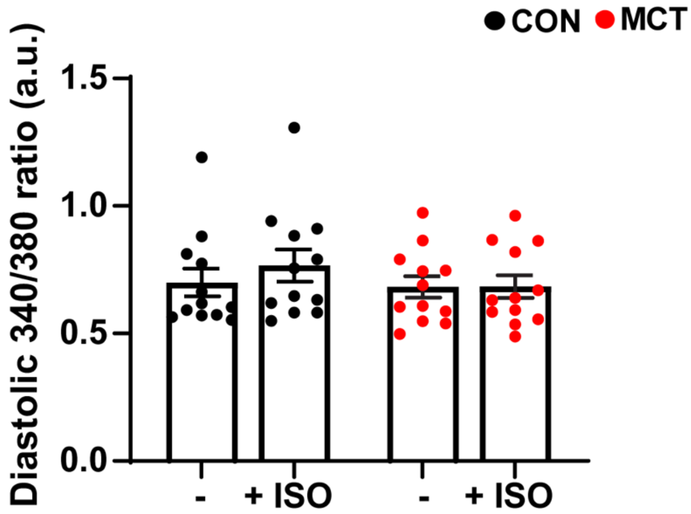

Supplementary Figure 1. Diastolic response to isoproterenol. Diastolic 340/380 ratio from the cytosolic Ca^{2+} response of control (CON, black) and monocrotaline (MCT, red) right ventricular (RV) cardiomyocytes to 1 µM isoproterenol. Myocytes were stimulated at 1 Hz and super-fused with 1 mM Ca^{2+} Tyrode's before (-) and during isoproterenol (+ ISO). Results are presented as mean ± SEM, n = 12 RV myocytes from N = 4 CON hearts, and n = 12 RV myocytes from N = 4 MCT hearts.

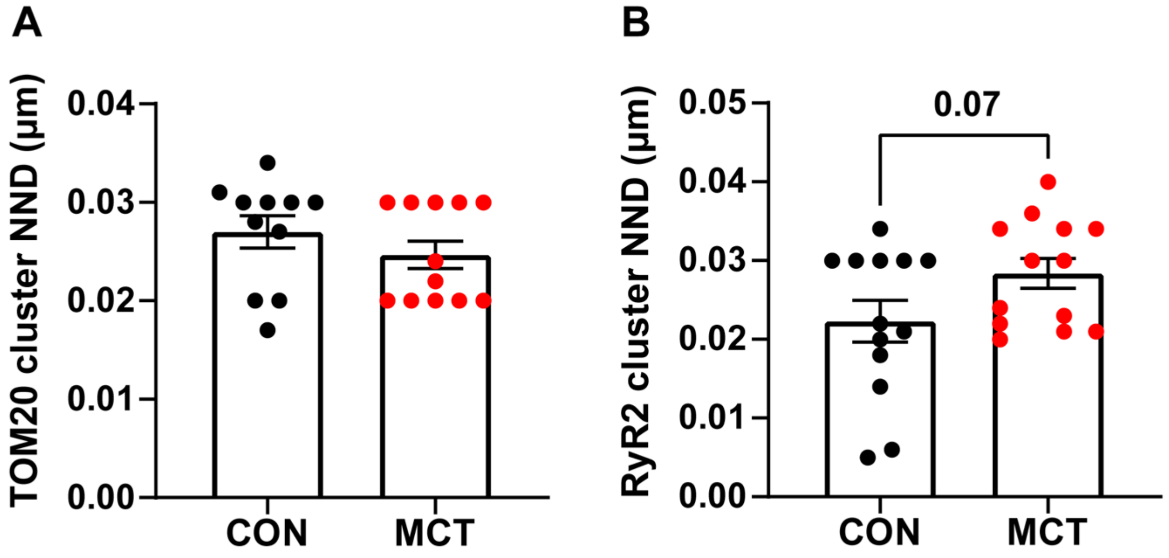

Supplementary Figure 2. Distances between clusters. Nearest neighbour distances between single RyR2 clusters and TOM20 clusters analysed from right ventricular (RV) tissue sections from control (CON, black) and monocrotaline (MCT, red) hearts, captured with stimulated emission depletion (STED) microscopy. Panels A and B show nearest neighbor distances (in µm) between single TOM20 clusters and single RyR2 clusters, respectively. Data are presented as mean ± SEM from N = 3 CON and N = 3 MCT hearts, and n = 3-5 RV sections/heart.
